# Targeted H_2_S-Mediated Gas Therapy with pH-Sensitive Release Property for Myocardial Ischemia–Reperfusion Injury by Platelet Membrane

**DOI:** 10.34133/bmr.0061

**Published:** 2024-08-19

**Authors:** Lin Liu, Yucen Yao, Yang Liu, Bingrong Hong, Ziqing Li, Xuejun Chen, Yaofeng Zhang, Hongbo Fu, Degong Yang, Chunrong Yang

**Affiliations:** ^1^ Department of Pharmacy, The Second Affiliated Hospital of Shantou University Medical College, Shantou 515041, China.; ^2^ Department of Pharmacy, Shantou University Medical College, Shantou 515041, China.; ^3^ College of Pharmacy, Jiamusi University, Jiamusi 154007, China.; ^4^ Department of Pharmacy, Department of Dermatology, The First Affiliated Hospital of Shantou University Medical College, Shantou 515041, China.

## Abstract

Management of myocardial ischemia–reperfusion injury (MIRI) in reperfusion therapy remains a major obstacle in the field of cardiovascular disease, but current available therapies have not yet been achieved in mitigating myocardial injury due to the complex pathological mechanisms of MIRI. Exogenous delivery of hydrogen sulfide (H_2_S) to the injured myocardium can be an effective strategy for treating MIRI due to the multiple physiologic functions of H_2_S, including anti-inflammatory, anti-apoptotic, and mitochondrial protective effects. Here, to realize the precise delivery and release of H_2_S, we proposed the targeted H_2_S-mediated gas therapy with pH-sensitive release property mediated by platelet membranes (PMs). In this study, a biomimetic functional poly(lactic-co-ethanolic acid) nanoparticle (RAPA/JK-1-PLGA@PM) was fabricated by loading rapamycin (RAPA; mTOR inhibitor) and JK-1 (H_2_S donor) and then coated with PM. In vitro observations were conducted including pharmaceutical evaluation, H_2_S release behaviors, hemolysis analysis, serum stability, cellular uptake, cytotoxicity, inhibition of myocardial apoptosis, and anti-inflammation. In vivo examinations were performed including targeting ability, restoration of cardiac function, inhibition of pathological remodeling, and anti-inflammation. RAPA/JK-1-PLGA@PM was successfully prepared with good size distribution and stability. Utilizing the natural infarct-homing ability of PM, RAPA/JK-1-PLGA@PM could be effectively targeted to the damaged myocardium. RAPA/JK-1-PLGA@PM continuously released H_2_S triggered by inflammatory microenvironment, which could inhibit cardiomyocyte apoptosis, realize the transition of pro-inflammation, and alleviate myocardial injury demonstrated in hypoxia/reoxygenation myocardial cell in vitro. Precise delivery and release of H_2_S attenuated inflammatory response and cardiac damage, promoted cardiac repair, and ameliorated cardiac function proven in MIRI mouse model in vivo. This research outlined the novel nanoplatform that combined immunosuppressant agents and H_2_S donor with the pH-sensitive release property, offering a promising therapeutic for MIRI treatment that leveraged the synergistic effects of gas therapy.

## Introduction

Acute myocardial infarction (MI) is a common manifestation of ischemic heart disease, becoming one of the leading causes of disability and death worldwide [1]. Timely and effective revascularization therapy of patients with MI through percutaneous coronary intervention can significantly reduce their acute mortality, but a large proportion of the patients eventually progress to heart failure [2]. Although reperfusion of ischemic tissue is vital for survival, it also initiates the secondary injury known as myocardial ischemia–reperfusion injury (MIRI). MIRI mainly comprises oxidative damage, cell death, and profound inflammatory immune response [3]. The underlying mechanisms of MIRI are complex and mainly related to mitochondrial dysfunction, intracellular calcium overload, oxidative stress damage, and inflammation cascade reaction [4,5]. As clinical outcomes remain insufficient, new therapeutic strategies are urgent to mitigate the above influences in MIRI tissues.

Hydrogen sulfide (H_2_S) has been found to be an important gaseous messenger involved in the cardiovascular system, along with nitric oxide (NO) and carbon oxide (CO) [6]. It plays an essential physiological role in inflammation, reperfusion injury, and circulatory shock of the cardiovascular and nervous systems. Specifically, H_2_S exerts its biological effects by generating multiple cytoprotective responses, including promoting of macrophage phenotype transition toward anti-inflammatory M2-type macrophage, relaxing vascularization, stimulating angiogenesis, and attenuating oxidative stress-related tissue injury [7–10]. Indeed, H_2_S has been proven to be a key “chemical stimulus” before and after ischemia, displaying its infarct-limiting effect [11,12]. As a gasotransmitter, H_2_S can rapidly trigger a variety of biological responses by traveling through cell membranes without utilizing specific transporters, whether it is endogenously produced or exogenously administered as H_2_S donor compounds [13,14]. However, many biological responses to H_2_S follow a bell-shaped dose dependence, ranging from low concentrations of physiological and cellular protective effects to high concentrations of cytotoxic effects [15]. Therefore, the H_2_S donors with controlled-release properties are essential to ensure no detriment to other tissues in the process of MIRI treatment. So far, several types of common H_2_S donors have been used [16]. Among them, sodium sulfide (Na_2_S) and sodium hydrogen sulfide (NaHS) are the 2 most common inorganic H_2_S-releasing compounds that exhibit the ability to rapidly increase the concentration of H_2_S. However, it is difficult to control the concentration of H_2_S due to the spontaneous release of H_2_S and its rapid volatilization [17–19]. In addition, synthetic H_2_S donors such as GYY4137 can slowly release H_2_S upon hydrolysis in water. But the hydrolysis of GYY4137 in pure water is too slow to be compromised [20]. To obtain the controllable release of H_2_S, Zhao et al. [17] employed JK-1 as a pH-dependent H_2_S donor for wound healing, which only produced H_2_S at acidic pH conditions. The pH response condition for H_2_S release from JK-1 was consistent with the inflammatory microenvironment in the process of MIRI. Since JK-1 is a small molecule, it is unsuitable to be applied to intravenous injection directly, urging the delivery system to prolong the H_2_S release and control its level in vivo. By incorporating H_2_S donors, serval types of H_2_S-containing biomaterials (hydrogels [21], nanoparticles [NPs] [22], nanoemulsions [23], etc.) have been developed to release H_2_S in a controllable manner in response to the biological environment. Therefore, we envisioned that JK-1 with pH-sensitive property could be applied to MIRI therapy through the in vivo advantage of H_2_S.

Inspired by the enhancement of circulating monocyte–platelet aggregates (MPAs) in MI patients, platelet (PLT) bionic technology has emerged as a potential navigation strategy for endowing nanoplatforms with actively targeting ischemic myocardium [24,25]. The binding between P-selectin and P-selectin glycoprotein ligand-1 (PSGL-1) is the main mediator of the interaction between PLTs and monocytes, relating to the severity of inflammation in MI [26,27]. Existing studies have shown that PSGL-1 is highly expressed in circulating Ly6C^+^ monocytes, which is the main source of inflammatory macrophages in the MI region. After MI, PLTs are activated early and bind to monocytes in blood vessels, further entering the ischemic myocardium. In addition, a considerable number of inflammatory Ly6C^high^ monocytes are recruited into the myocardium and then differentiated into macrophages [28]. Therefore, simulating the interaction between PLTs and monocytes may enhance the targeted delivery of the drug delivery system to the ischemic myocardium via preferentially binding to monocytes in circulation. Considering the increased binding ability of PLTs to monocytes in circulation after MIRI, platelet membrane (PM) is introduced for targeted drug delivery therapy in cardiac repair [29]. Based on the natural infarct-homing ability of PLTs, we assumed that the PM-encapsulated nanoplatforms could bind to Ly6C^high^ monocytes in circulation and be carried by monocytes into the ischemic myocardium.

**Fig. 1.**
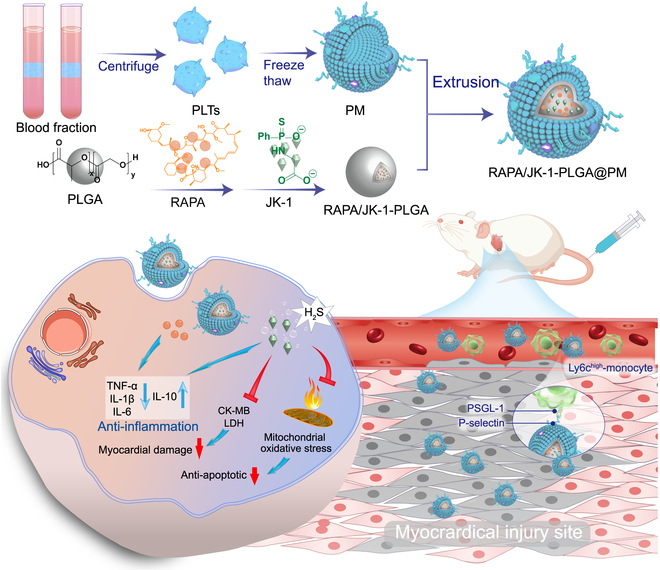
Schematic illustration of the myocardial injury-targeting NPs with pH-sensitive release property for JK-1 and RAPA co-delivery for the gas therapy of MIRI. The biomimetic engineered NPs were developed to envelop JK-1 and RAPA-loaded PLGA NPs and encapsulate PM. After intravenous injection to rats experiencing MIRI, RAPA/JK-1-PLGA@PM could be efficiently delivered into the myocardial injury due to the natural infarct-homing ability of PM. JK-1 continuously released H_2_S triggered by inflammation microenvironment, which could inhibit cardiomyocyte apoptosis and simultaneously realize the transition of pro-inflammation. Thus, H_2_S and RAPA cooperated with anti-inflammation efficacy, which ultimately contributed to the pronounced inhibition of myocardial injury and recovery of myocardial function.

In this context, we fabricated engineered biomimetic NPs by encapsulating JK-1 and rapamycin (RAPA) in biocompatible poly(lactic-co-ethanolic acid) (PLGA) shells coated with PM (Fig. 1) [30,31]. PLGA was applied in drug delivery systems due to its ability to both protect the H_2_S donor from degradation and sustain the release of the H_2_S donor. After intravenous administration, RAPA/JK-1-PLGA@PM could target ischemic myocardial tissue through the natural infarct-homing ability of PM, followed by the effective release of JK-1 and RAPA, together improving MIRI. As a mammalian target of RAPA (mTOR) inhibitor, RAPA reduced myocardial fibrosis and exerted anti-inflammatory effects to alleviate cardiac dysfunction. More importantly, the released JK-1 continuously released H_2_S in response to the inflammatory acidic pH condition, which could inhibit the production of inflammatory cytokines and cardiomyocyte apoptosis, stimulate angiogenesis, attenuate oxidative stress-induced myocardial injury, and ultimately realize the therapeutic effect. This work elucidated the design of engineered biomimetic NPs based on gas therapy and their therapeutic potential in MIRI. Such an effective and biocompatible cardioprotective strategy highlighted the therapeutic prospects for patients benefiting from MIRI.

## Materials and Methods

### Materials

RAPA and polyvinyl alcohol (PVA) were purchased from Aladdin (Shanghai, China). PLGA was supplied by Daigang Bio (Shandong, China). Heparin was bought from Cenbest (Nanjing, China). The platelet extraction kit and ADC anticoagulant were purchased from Shanghai Huzhen Bio (Shanghai, China). JK-1 was gifted by X. Zhao (Shenyang Pharmaceutical University). Fetal bovine serum (FBS), trypsin, and CCK-8 assay kit were obtained from Dalian Meilun Biotechnology (Dalian, China). Mouse lactic dehydrogenase (LDH) enzyme-linked immunosorbent assay (ELISA) kit, mouse creatine kinase isoenzyme (CK-MB) ELISA kit, mouse interleukin-10 (IL-10) ELISA kit, mouse IL-6 ELISA kit, mouse tumor necrosis factor-α (TNF-α) ELISA kit, and mouse IL-1β ELISA kit were bought by Elabscience Biotechnology Co. Ltd. (Wuhan, China). Isoflurane was bought from Ruiwode Life Technology Co. Ltd. (Shenzhen, China). All chemical and biological reagents were of analytical grade or above.

### Animals and cell culture

Male C57BL/6 mice (18 to 22 g) were supplied by the Experimental Animal Center of Shantou University (Guangdong, China). Procedures performed on animals conformed to the *Guide for the Care and Use of Laboratory Animals* published by the National Institutes of Health (NIH) and were approved by the Institutional Animal Research and Use Committee of Shantou University (SUMC2022-152).

Rat cardiomyocyte (H9C2) cells were purchased from the Cell Bank of the Chinese Academy of Sciences. Cells were cultured with high-glucose basic Dulbecco’s modified Eagle’s medium (DMEM) containing 10% FBS and 1% penicillin-streptomycin solution (10,000 U ml^−1^) at 37°C, 5% CO_2_ in the incubator.

### Isolation and quantification of PM

The PM was isolated from C57BL/6J mice through repeated freeze–thaw method as previously described with modification [3]. In brief, the mice plasma was centrifuged at 100*g* for 20 min. The isolated PLTs were then pelleted by centrifugation at 800*g* for 20 min at room temperature. The PM fragments were obtained by 3 freeze–thaw cycles. The PM was injected into the Avanti mini extruder (Avanti Polar Lipids, Alabaster, AL, USA) and repeatedly extruded through polycarbonate porous membranes; thus, the PM was fabricated. The PM was preserved at −80°C until further use. The yield of isolated PM was approximately 29.45 ± 3.84 μg proteins per 10 ml as estimated by BCA assay.

### Preparation of RAPA/JK-1-PLGA@PM

RAPA (1 mg) and PLGA (20 mg) were dissolved in 1 ml of CH_2_Cl_2_ and slowly dropped into 10 ml of 1% PVA aqueous solution containing JK-1 under magnetic stirring. After ultrasound (270 W), the organic solvent was removed by vacuum evaporation at 60°C, and RAPA/JK-1-PLGA NP solution (RAPA/JK-1-PLGA) was prepared by filtration with a microporous membrane (0.22 μm). Subsequently, NP solution (1 μg/ml) and PM (0.5 μg/ml) were mixed and sonicated for 10 min at 37°C and 100 W. The PM-encapsulated NPs (RAPA/JK-1-PLGA@PM) were obtained by squeezing the mixture with a micro-extruder.

### Characterization of RAPA/JK-1-PLGA@PM

The size distribution and zeta-potential were measured using dynamic light scattering (DLS) (Zetasizer Nano, Malvern, UK). The morphology was observed through transmission electron microscopy (TEM; Hitachi, Japan). In short, diluted NPs were added dropwise onto a copper mesh. After retaining NPs for 40 s, the copper mesh was dried at room temperature and NPs were observed under TEM. To measure the encapsulation efficiency (*EE%*) and loading capacity (*LC%*) of RAPA, the NPs and unencapsulated RAPA were separated by ultrafiltration (molecular weight cut-off: 3 kDa, 12,000 rpm, 10 min). The filtrate was collected into a volumetric flask, diluted to a volume with methanol, and sonicated for 20 min, followed by filtering using a 0.22-μm membrane. The concentration of RAPA was determined by ultraviolet (UV) spectrophotometry at 278 nm. The calculation was as follows:

$$EE\%=\frac{\text{weight of encapsulated RAPA}}{\text{weight of RAPA added}}\times 100\%$$

$$LC\%=\frac{\text{weight of encapsulated RAPA}}{\text{weight of}\;NPs}\times 100\%$$

### Serum stability and long-term placement stability

NPs were resuspended to phosphate-buffered saline (PBS) solution (containing 10% FBS, pH 7.4) to assess the serum stability. The final concentration was 2.4 mg/ml (equal to the administration dose in vivo). Particle size as an evaluation index was measured. To evaluate the long-term placement stability, NPs were placed, and the particle sizes were measured at 0, 1, 2, and 3 months.

### Western blotting assay

The integrity of PM proteins was investigated by measuring the amount of biomarker proteins on PM using Western blot analysis. Briefly, PM or RAPA/JK-1-PLGA@PM containing an equivalent quantity of total proteins was extracted using sodium dodecyl sulfate–polyacrylamide gel electrophoresis (SDS-PAGE) and transferred to a polyvinylidene difluoride (PVDF) membrane. Then, the PVDF membrane was incubated overnight with primary antibodies. On the second day, the mixture was incubated with secondary antibodies for 4 h. The target proteins were visualized by chemical immunofluorescence after adding the developer (ChemiDoc XRS, Bio-Rad, Hercules, CA, USA). Protein expression was quantified by densitometry analysis using the ImageJ software (NIH, USA).

### Red blood cell hemolysis

Red blood cell (RBC) suspension (5%) was mixed with the samples (containing RAPA/JK-1-PLGA@PM), reaching a final concentration of 200, 500, and 700 μg/ml. The RBC suspension treated with saline and distilled water was used as the positive and negative control, respectively. Incubated at 37°C for 3 h and centrifuged for 15 min. The obtained precipitate was used for RBC hemolysis analysis.The absorbance of the supernatant was measured at 540 nm. The hemolysis rate was calculated according to the following equation:

$$\text{Hemolysis rate}=\frac{\left(A_{H}-A_{0}\right)}{\left(A_{D}-A_{0}\right)}\times 100\%$$

where *A*_*H*_ is the absorbance value of the NP dispersion, *A*_*D*_ is the absorbance value of distilled water, and *A*_*0*_ is the absorbance value of 0.9% NaCl solution.

### In vitro drug and H_2_S release from NPs

The in vitro release behavior of RAPA from different preparations was performed by the dynamic dialysis method. After being enclosed into the dialysis bag, the preparations (4 ml) were added to PBS (400 ml, pH 6.8) at 37 ± 0.1°C, stirring at 100 rpm continuously. At presetting time points, 1 ml of the sample was withdrawn for detection and replaced with an equal volume of dissolving media that had preheated. The RAPA concentration was determined by UV–vis spectrophotometer.

The release of H_2_S from NPs was determined by the methylene blue colorimetric method. The dialysis bag containing freshly prepared NP solution (50 mM JK-1) was placed in PBS (pH 6.5, 7.4), inducing the donor to produce H_2_S. Freshly prepared JK-1 solution (50 mM) served as a control. The release medium (1.0 ml) was divided at preset time points and transferred to centrifuge tubes containing zinc acetate (1%, w/v) and NaOH solution (1.5 M). After centrifugation for 20 min, the supernatant was removed, and the zinc sulfide pellet was reconstituted with *N*,*N*-dimethyl-1,4-phenylenedimine sulfate (20 mM in 7.2 M HCl) and ferric chloride (30 mM in 1.2 M HCl). After 20 min of methylene blue reaction, the absorbance was measured at 675 nm using a microplate reader (Bio-Rad Laboratories, Hertfordshire, UK). The H_2_S concentration of each sample was calculated against a calibration curve obtained by a series of Na_2_S solutions.

### In vitro cytotoxicity

Cytotoxicity for different formulations and blank NPs was examined by the CCK-8 assay kit. Briefly, H9C2 cells were cultured in 96-well plates at a density of 1 × 10^4^ cells per well overnight. The medium was replaced with fresh medium containing different concentrations of free RAPA, RAPA-PLGA, RAPA/JK-1-PLGA, and RAPA/JK-1-PLGA@PM for 24 h. The cells were treated with CCK-8 dilution (10%) for 4 h, and then the cell viability was measured at 450 nm by a microplate reader.

### In vitro cellular uptake

The internalization of different formulations was investigated using laser confocal microscopy (CLSM) and flow cytometry. Coumarin-6 (C6) was used as a fluorescent probe instead of RAPA [32]. H9C2 cells were seeded in 12-well plates and incubated at 37°C for 12 h. The medium was replaced with a fresh medium containing fluorescence-labeled formulations. After 1 and 4 h of incubation, the cells were washed with PBS and incubated with 4% paraformaldehyde and counterstained with Hoechst 33258. Cells were then thoroughly washed with PBS and observed by the LSM 880 Laser Scan Microscope (Carl Zeiss AG, Jena, Germany). Subsequently, the cells were collected with trypsin and cellular uptake was quantified by flow cytometry (BD Biosciences, USA).

### Establishment of cardiomyocyte hypoxia/reoxygenation model

H9C2 cells were seeded on a 6-well plate at a density of 1 × 10^6^ cells per well and cultured at 37°C for 24 h. After being replaced with DMEM without FBS, the cells were placed in a hypoxic workstation for hypoxia (1% O_2_–5% CO_2_, 37°C). Subsequently, the cells were reoxygenated in a conventional cell culture incubator in a DMEM containing 10% FBS to establish an H9C2 cardiomyocyte hypoxia/reoxygenation (H/R) injury model. The optimal H/R time for the H/R injury model was determined by screening the hypoxia time.

### In vitro cell apoptosis

The annexin V–fluorescein isothiocyanate (FITC)/propidium iodide (PI) apoptosis assay kit was used to evaluate the anti-apoptotic ability of different NPs on H/R cardiomyocytes. After H/R, H9C2 cells were incubated with RAPA/JK-1-PLGA@PM together for 24 h. Subsequently, cells were collected, washed with PBS, stained with annexin V–FITC/PI, and finally measured by flow cytometry. In addition, cell viability was measured to evaluate the anti-apoptotic ability of different NPs on H/R cardiomyocytes.

### Measurement of cardiomyocyte injury levels in vitro

To investigate the injury degree of the cardiomyocytes after different treatments, an ELISA was performed to measure the levels of indicators of myocardial injury including creatine kinase isoenzyme (CK-MB) and lactate dehydrogenase (LDH). After hypoxia culture, H9C2 cells were replaced in DMEM containing free RAPA, RAPA-PLGA, RAPA/JK-1-PLGA, and RAPA/JK-1-PLGA@PM for reoxygenation. After centrifugation, the supernatant was removed, the CK-MB concentration was determined using the mouse CK-MB ELISA kit, and the LDH concentration was determined using the LDH detection kit. After adding the stop solution, the UV absorbance value at 450 nm was measured using a microplate reader.

### Measurement of the anti-inflammation ability in vitro

The capability to reduce extracellular cytokine levels of RAPA/JK-1-PLGA@PM after incubation with H/R cardiomyocytes has been investigated. Briefly, H9C2 cells were inoculated in 96-well plates (1 × 10^5^ cells per well) and cultured overnight. After hypoxia, the cells were co-incubated with various preparations and subjected to reoxygenation treatment. Then, concentrations of cytokines (TNF-α, IL-1β, IL-6, and IL-10) were measured by ELISA.

### Establishment of MIRI model

Surgery induction of cardiac ischemia/reperfusion (I/R) was subjected to C57BL/6 mice as in a previous study with slight modifications. Briefly, all mice were anesthetized with 1% pentobarbital sodium. The mice were mechanically ventilated with 110 breaths per minute and a tidal volume of 2 ml. The electrocardiograms were observed through a multi-channel physiological signal acquisition and processing system. The third intercostal space above the left chest and heart was exposed. After the left thoracotomy, the left anterior descending coronary artery was with an 8-0 silk ligature tied with a slip knot. After 30 min of ischemia, the slip knot was released by pulling the outside end of the suture smoothly, allowing for myocardial reperfusion. The chest cavity was closed, sutured, and disinfected for subsequent experiments.

### Echocardiographic analyses of cardiac function

The cardiac function of mice treated with different preparations was evaluated on the 7th day after MIRI surgery. The mice were anesthetized by inhalation of isoflurane using a gas anesthesia system. Then, the mice were anesthetized with inhaled isoflurane, maintaining a heart rate of around 400 to 500 beats per minute. Using a small-animal ultrasound system for cardiac ultrasound detection, the long- and short-axis sections of the left sternum of mice were placed on the MS-400 ultrasound probe to collect B-mode and M-mode images. The average of 3 cardiac cycles was taken as the cardiac function parameter. Accordingly, cardiac parameters such as left ventricular ejection fraction (LVEF) and left ventricular shortening fraction (LVFS) were determined.

### Biodistribution of NPs in vivo

The distribution of RAPA/JK-1-PLGA@PM in mice was performed through in vitro fluorescence imaging. Referring to relevant literature, C6 was used to replace RAPA. After injecting an equal amount of NP solution into the tail vein, the mice were euthanized 24 h later. The main organs (heart, liver, spleen, lungs, and kidney) of the mice were used for in vitro fluorescence imaging via the FX Pro imaging system (Bruker Inc., USA).

### Measurement of myocardial injury in vivo

To investigate the myocardial injury after different treatments, ELISA was performed to measure the levels of CK-MB and LDH. After 7 days of modeling, the blood of the retro-orbital plexus was collected. After centrifugation, the supernatant was removed and the concentrations of CK-MB and LDH were measured at 450 nm using a microplate reader.

### Measurement of the anti-inflammation ability in vivo

The anti-inflammatory ability was evaluated by examining the serum cytokine levels after different treatments. After 7 days of surgery, the blood of the retro-orbital plexus was collected. After centrifugation, the supernatant was removed and the cytokine concentrations of TNF-α, IL-1β, IL-6, and IL-10 were measured.

### Histological analysis

The mice were anesthetized via intraperitoneal injection with 20% urethane (60 μl/100 g) on the 7th day after MIRI surgery. After thoracotomy, the hearts were fixed with trans-cardiac perfusion of saline and immersed in 4% paraformaldehyde for 24 h. Heart tissue samples were dehydrated with gradient ethanol and xylene and embedded in paraffin blocks, cutting into slices with a thickness of 5 μm. Paraffin sections were stained with Masson trichrome and hematoxylin-eosin (H&E). Finaly, the sections were observed using a digital pathology scanner (Ventana iScan Coreo, USA).

### Statistical analysis.

All the data were shown as means ± SD. One-way analysis of variance (ANOVA) analysis was applied to determine the significance of the difference. Statistical significance was defined as *P < 0.05, **P < 0.01, and ***P < 0.001.

## Results

### Preparation and characterization of RAPA/JK-1-PLGA@PM

**Fig. 2.**
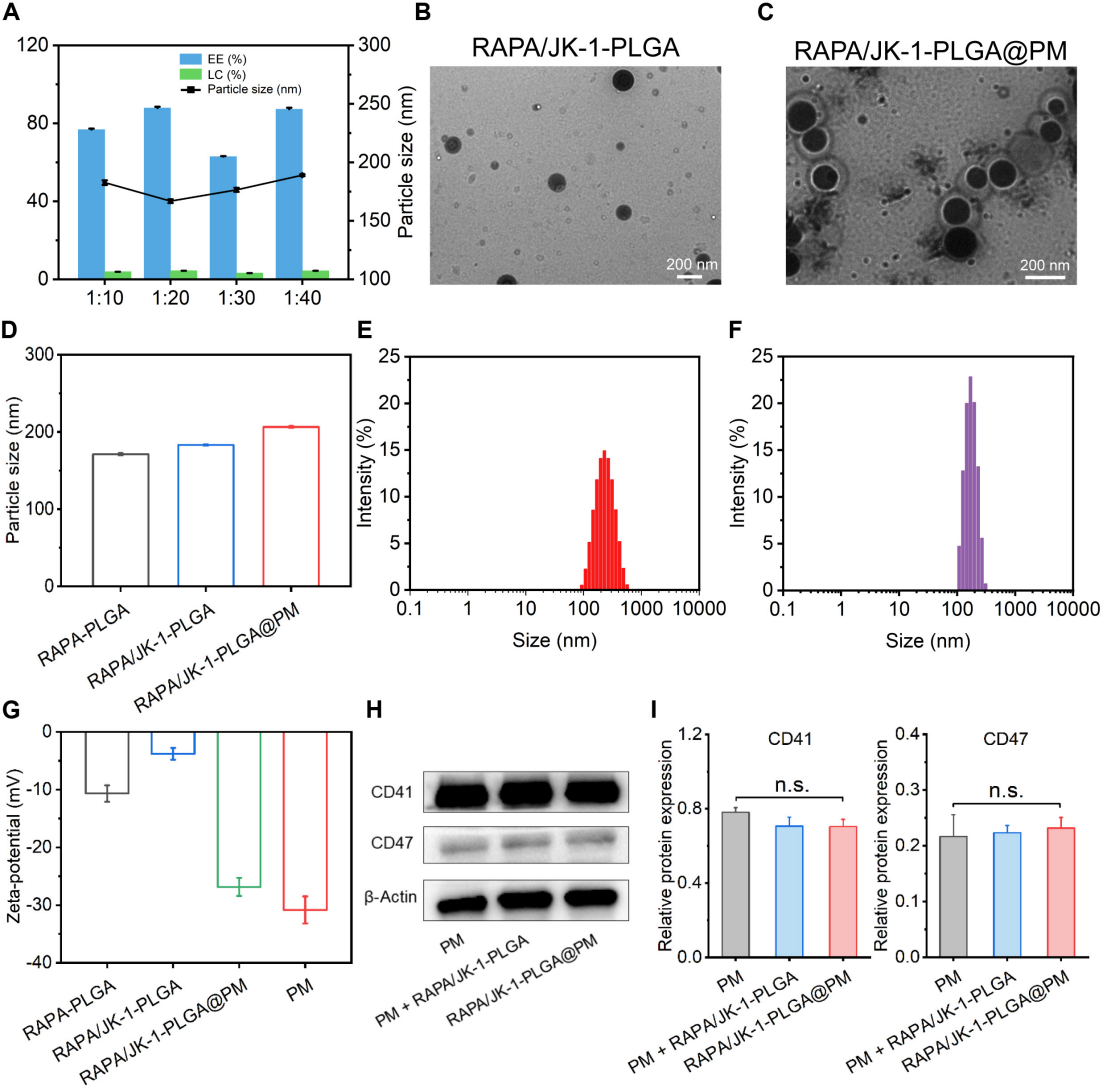
Characterization of RAPA/JK-1-PLGA@PM. (A) EE%, LC%, and particle sizes of NPs formed with diverse ratios (w/w) of RAPA and blank carrier. TEM images of RAPA/JK-1-PLGA (B) and RAPA/JK-1-PLGA@PM (C). (D) Particle size of RAPA-PLGA, RAPA/JK-1-PLGA, and RAPA/JK-1-PLGA@PM. Particle size distribution of RAPA/JK-1-PLGA (E) and RAPA/JK-1-PLGA@PM (F). (G) Zeta-potential of RAPA-PLGA, RAPA/JK-1-PLGA, RAPA/JK-1-PLGA@PM, and PM. (H and I) Serum stability and long-term placement stability of NPs. Data were shown as means ± SD (*n* = 3). n.s., not significant.

RAPA/JK-1-PLGA was obtained using the ultrasonic emulsification method. The ratio of RAPA to the carrier was chosen as 1:20 (w/w), which had the high EE% (87.78%), LC% (4.39%), and the suitable particle size for systemic circulation (Fig. 2A). Then, membrane-coated NPs were fabricated by the co-extrusion method. TEM images displayed the successful fabrication of RAPA/JK-1-PLGA with the regularly spherical-shaped morphology and RAPA/JK-1-PLGA@PM with the obvious core–shell structure (Fig. 2B and C). As illustrated in Fig. 2D to F, the average particle size of RAPA-PLGA, RAPA/JK-1-PLGA, and RAPA/JK-1-PLGA@PM was 171.11, 183.1, and 206.5 nm, respectively, with a narrow polydispersity index (PDI), indicating the high dispersion properties. The encapsulation of PM led to an augmentation of particle size of about 20 nm, consistent with TEM images. Furthermore, the zeta-potential of RAPA/JK-1-PLGA@PM was distinctly decreased owing to the properties of PM, which also proved the successful encapsulation of PM. The surface zeta-potential of RAPA/JK-1-PLGA@PM was close to that of the PM (−30.84 mV) (Fig. 2G). The negative surface charge made RAPA/JK-1-PLGA@PM less likely to be swallowed by the reticuloendothelial system, thereby prolonging their circulation time in vivo [33]. The serum stability of NPs was explored for 4 days of incubation with 10% serum solution (Fig. 2H). The long-term placement stability of NPs was evaluated for 3 months at 4 ± 2°C (Fig. 2I). Distinct change in particle size was not found during 2 stability experiments, indicating that NPs presented good stability.

**Fig. 3.**
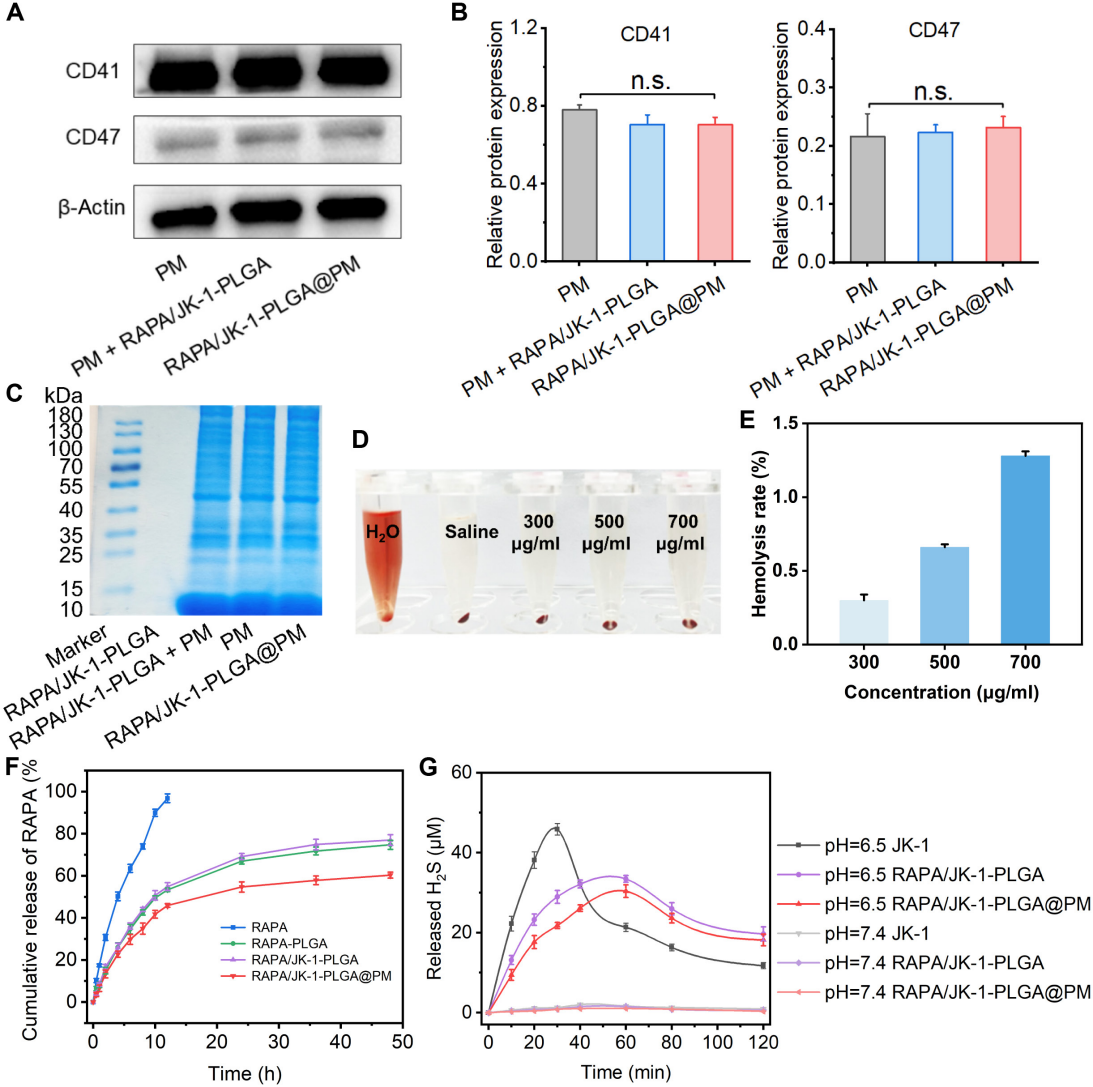
(A and B) Western blot assay of the CD41 and CD47 protein expression. (C) Coomassie blue assay was performed to detect the protein of PM. (D and E) Hemocompatibility evaluation of RAPA/JK-1-PLGA@PM in vitro*.* (F) Cumulative RAPA release from different preparations in PBS. (G) Concentrations of H_2_S released from different NPs in PBS (pH 6.5 and 7.4). Data were shown as means ± SD (*n* = 3).

Next, Western blotting was conducted to analyze specific protein markers (CD41 and CD47) [34]. The results showed that RAPA/JK-1-PLGA@PM retained the surface proteins inherited from the source cells (Fig. 3A and B). These above results confirmed the successful preparation of RAPA/JK-1-PLGA@PM. Furthermore, SDS-PAGE confirmed that most of the proteins on PM had been successfully modified to the surface of NPs (Fig. 3C). They have good biocompatibility and better targeting properties. Since RAPA/JK-1-PLGA@PM was designed to be delivered by intravenous administration, the blood compatibility was investigated in terms of thromboresistance. RBC hemolysis study was performed to investigate the hemolysis of RAPA/JK-1-PLGA@PM. As displayed in Fig. 3D and E, safety and great biocompatibility were exhibited in RAPA/JK-1-PLGA@PM, because the hemolysis rate was 1.28 ± 0.03% even at the high concentration (700 μg/ml), based on the hemolytic ratio and photograph. The stability of NPs in serum was a critical parameter for systemic administration.

In vitro drug release profile of RAPA/JK-1-PLGA@PM was performed by dynamic dialysis method in the acidic environment of inflammatory conditions. As shown in Fig. 3F, the results demonstrated that RAPA rapidly released from RAPA/JK-1-PLGA@PM in the initial 10 h and gradually released within 48 h. The cumulative release percentage of the drug from RAPA/JK-1-PLGA@PM reached about 61% within 48 h. The burst release of drug was not observed, indicating that RAPA/JK-1-PLGA@PM possessed good stability in vivo, which was conductive to systemic circulation.

Subsequently, the H_2_S release behaviors from RAPA/JK-1-PLGA@PM were measured at different pH values. As shown in Fig. 3G, the H_2_S from the free JK-1, RAPA/JK-1-PLGA, and RAPA/JK-1-PLGA@PM was released prominently in the weakly acidic pH condition. In comparison, there were almost no H_2_S release in the normal physiological environment (pH 7.4), displaying the pH-dependent H_2_S release manner. The content of H_2_S in the solution decreased continuously with time, due to the limited solubility of H_2_S. As the concentration increased, H_2_S continued to escape into the air. In addition, free JK-1 released almost no H_2_S at pH 7.4, whereas it released the most H_2_S at pH 6.5. This suggested that JK-1 had the property of pH-responsive release of H_2_S and that encapsulation by NPs delayed the release of JK-1, thus prolonging the H_2_S release profile of JK-1. Therefore, in combination with the dynamic changes in myocardial tissue pH during MIRI, this pH-sensitive H_2_S release property of JK-1 made the designed NPs ideal for the treatment of MIRI. Briefly, the inflammatory cascade response after the onset of MI triggered the rapid release of H_2_S from JK-1, which exerted its therapeutic effects. As the MIRI healing process progressed, the pH gradually increased to the weakly alkaline range under normal physiological conditions, allowing JK-1 to release H_2_S smoothly and gently [35].

### Cellular uptake and cytotoxicity assay

**Fig. 4.**
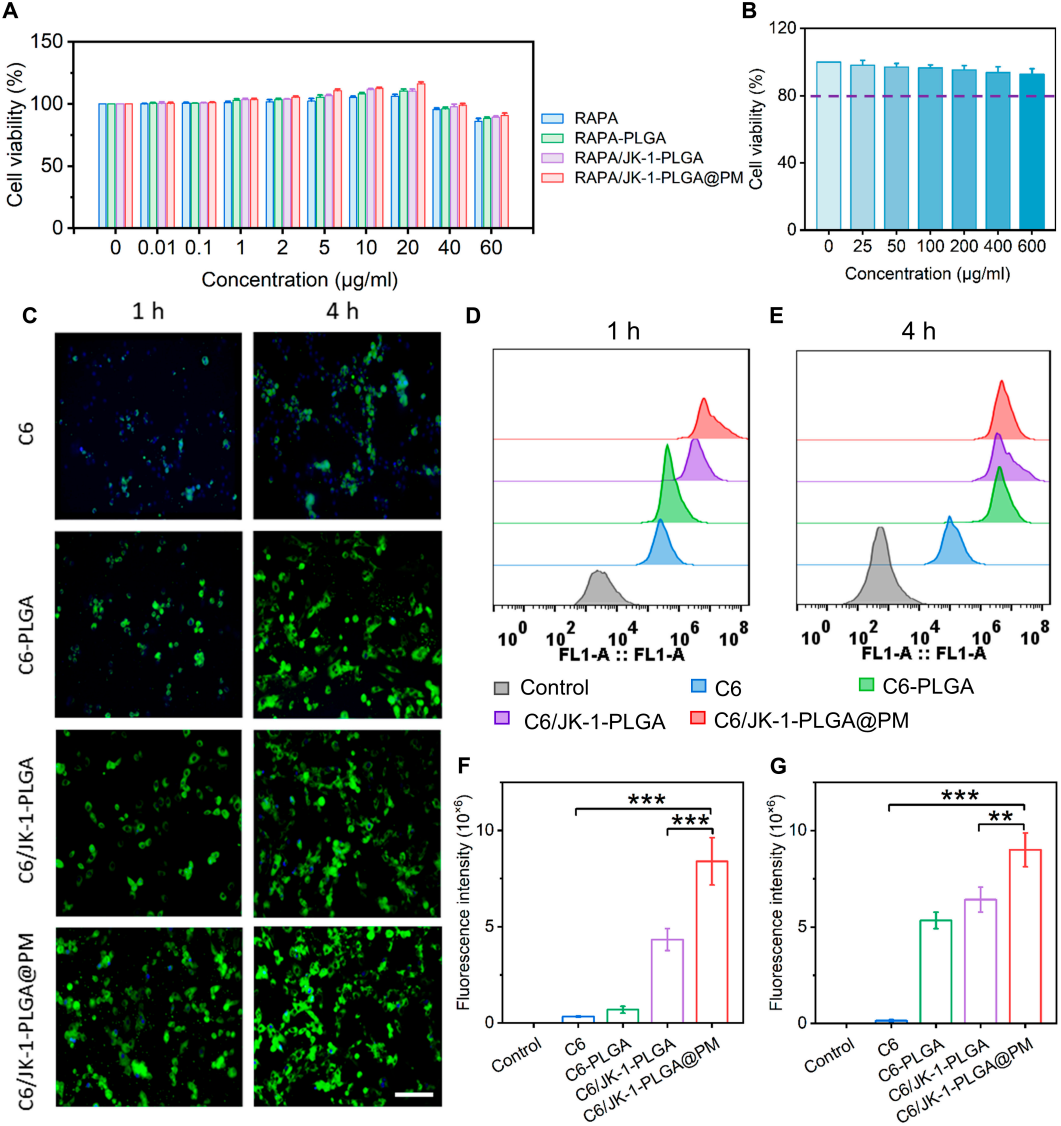
Cellular uptake and cytotoxicity of different NPs in H9C2 cells. (A) Cell viability of H9C2 cells treated with different preparations at various concentrations. (B) Cell viability of H9C2 cells treated with blank NPs at various concentrations. (C) CLSM images of different NPs with fluorescent labels in H9C2 cells. For observation by CLSM, nuclei were stained with 4′,6-diamidino-2-phenylindole (DAPI) (blue), while the preparations were stained with FITC (green). Scale bar, 50 μm. After incubation in H9C2 cells for 1 and 4 h, flow cytometry (D and E) and quantitative (F and G) results of treatment with different NPs. Statistical analysis was performed using one-way ANOVA. When ***P < 0.001, **P < 0.01, and *P < 0.05, the difference was statistically significant. Data were shown as means ± SD (*n* = 3).

Cellular internalization was a prerequisite for nanomedicine to exert the effects via intracellular pathways [36,37]. At the outset, cytotoxicity of blank NPs and different preparations was evaluated in H9C2 cells through CCK-8 assay. Negligible cytotoxicity could be observed for all groups after incubation with various preparations (Fig. 4A and B), indicating that these preparations possessed good biosafety.

CLSM and flow cytometry examined the capability of diverse preparations on cellular uptake. RAPA was replaced by C6 as a fluorescence probe. After fluorescent-labeled NPs were incubated with H9C2 cells, observation by CLSM revealed time-dependent internalization of RAPA/JK-1-PLGA@PM in H9C2 cells (Fig. 4C). It was noted that RAPA/JK-1-PLGA@PM displayed apparently stronger fluorescent intensity than the NPs of uncoating PM. The above result based on fluorescence observation was further demonstrated by quantification of intracellular fluorescent intensity via flow cytometry analysis. As displayed in Fig. 4D to G, RAPA/JK-1-PLGA@PM could be efficiently internalized by H9C2 cells. Consistent results were obtained in terms of the mean fluorescence intensity (MFI). The MFI of RAPA/JK-1-PLGA@PM was 1.94-fold higher at 1 h, compared to RAPA/JK-1-PLGA, indicating that PM played a vital role during the cellular uptake process. These findings observed that PM modification facilitated the targeting of cardiomyocytes and enhanced the intracellular uptake of RAPA/JK-1-PLGA@PM. Hence, RAPA/JK-1-PLGA@PM could be effectively internalized by cardiomyocytes and was relatively safe for MIRI therapy.

### Evaluation of anti-apoptosis ability in vitro

**Fig. 5.**
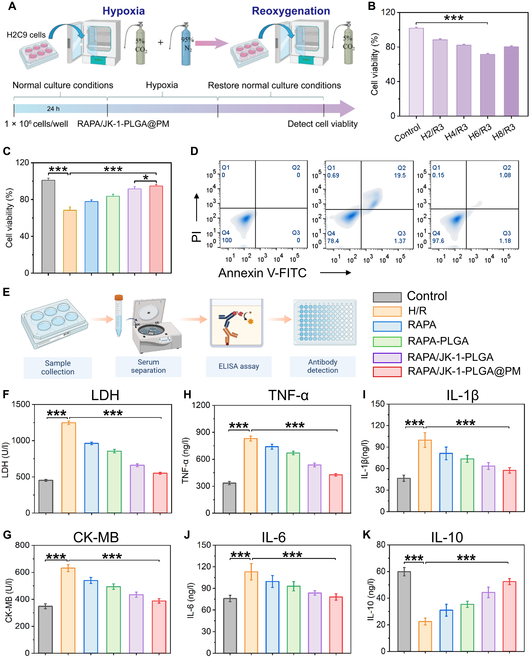
(A) Schematic diagram of H/R myocardial cell model building. (B) Cell viability of H/R H9C2 cells of different periods of hypoxia. (C) Cellular viability of H/R myocardial cells after different treatments. (D) Flow cytometry profiles of apoptotic H/R cardiomyocytes after different treatments. (E) ELISA experimental procedure flowchart. Cardiac levels of CK-MB (F) and LDH (G) in H/R cardiomyocytes. Extracellular cytokine levels of TNF-α (H), IL-1β (I), IL-6 (J), and IL-10 (K) in H/R cardiomyocytes. Statistical analysis was performed using one-way ANOVA. When ***P < 0.001, **P < 0.01, and *P < 0.05, the difference was statistically significant. Data were shown as means ± SD (*n* = 3).

The rescue of injured cardiomyocytes was a key issue in repairing infracted myocardium. To investigate the inhibitory effect of RAPA/JK-1-PLGA@PM on myocardial injury, H9C2 cells were subjected to H/R treatment to simulate I/R injury in vitro and cocultured with NPs to measure cell damage (Fig. 5A). After cell attachment, anaerobic conditions were used for cell incubation for ischemia induction. Afterward, the cells were incubated under normoxic conditions to trigger reperfusion. By adjusting the ischemic treatment time, we found that the treatment of 6-h ischemia and 3-h reperfusion effectively made 28.42% of H9C2 cell apoptosis (Fig. 5B). Then, we optimized the period to 6 h (ischemia) + 3 h (reperfusion) for the upcoming H/R strategy to simulate in vitro I/R injury.

The therapeutic effect of RAPA/JK-1-PLGA@PM was proven with the H/R-injured cardiomyocytes via CCK-8 assay and flow cytometry. H9C2 cells were incubated with different preparations and performed with H/R treatment. It was discovered that RAPA/JK-1-PLGA@PM could effectively reduce intracellular oxidative damage. The cell viability of H2C9 cells was significantly increased by treatment with RAPA/JK-1-PLGA@PM (94.76%), compared to the H/R group (68.32%) (Fig. 5C). The cell apoptosis detection of flow cytometry provided similar results (Fig. 5D). With the treatment of RAPA/JK-1-PLGA@PM, the apoptosis rate decreased from 19.5% to 1.08%. These results also suggested the anti-apoptotic capability of RAPA/JK-1-PLGA@PM for the damaged myocardium. The better therapeutic effect of RAPA/JK-1-PLGA@PM could be ascribed to the protective effect on the damaged myocardium of the released H_2_S from JK-1, which maintained mitochondrial function and inhibited myocardial cell apoptosis. As an antioxidant, H_2_S was exhibited to alleviate the state of oxidative stress by scavenging excess reactive oxygen species (ROS) produced during the H/R process, as well as promoting the expression of antioxidant genes such as heme oxygenase (HO-1) in cardiomyocytes and inhibiting ROS production in mitochondria through p66shc-dependent signaling [38]. In addition, the phenomenon could also be taken by the synergistic effect of RAPA and H_2_S after loading by NPs.

### Inhibition of myocardial injury ability in vitro

Serum creatine kinase isoenzyme (CK-MB) and lactate dehydrogenase (LDH) were widely recognized as potential biomarkers to assess the ischemic severity of myocardial injury, which were released from the cytosol of cardiomyocytes into the blood following myocardial injury [39,40]. The extracellular levels of LDH and CK-MB were measured to evaluate the inhibitory effect of NPs on myocardial injury by ELISA (Fig. 5E). After treatment with H/R injury, H9C2 cells produced higher levels of CK-MB and LDH, indicating that H/R treatment could cause severe myocardial injury. Surprisingly, the levels of LDH and CK-MB in H/R myocardial extracellular matrix were all significantly decreased in the RAPA/JK-1-PLGA@PM group, compared to the H/R group (Fig. 5F and G). Furthermore, to investigate the anti-inflammatory activity of different preparations in vitro, we evaluated the levels of inflammatory cytokines by H/R-induced H9C2 cells. ELISA in the H/R group showed that the expression levels of TNF-α and IL-1β increased significantly, and the expression of IL-6 and IL-10 drastically decreased (*P* < 0.001). RAPA-PLGA, RAPA/JK-1-PLGA, and RAPA/JK-1-PLGA@PM groups could significantly reverse the pathological changes of inflammatory and anti-inflammatory factors after 24-h incubation. Among them, the reversal effect of the RAPA/JK-1-PLGA@PM group was more prominent and statistically significant (*P* < 0.001) (Fig. 5H to K). The significant anti-inflammatory effects were attributed to precise release of H_2_S. The above results showed that RAPA/JK-1-PLGA@PM possessed favorable inhibitory myocardial injury and inflammatory properties, owing to significant anti-apoptosis and attenuation effects of pro-inflammatory factors by H_2_S [10,41]. Afterward, the therapeutic efficacy of the myocardium-targeted nanoplatform based on gas therapy was further explored on MIRI mouse models.

### In vivo efficacy and targeting studies

**Fig. 6.**
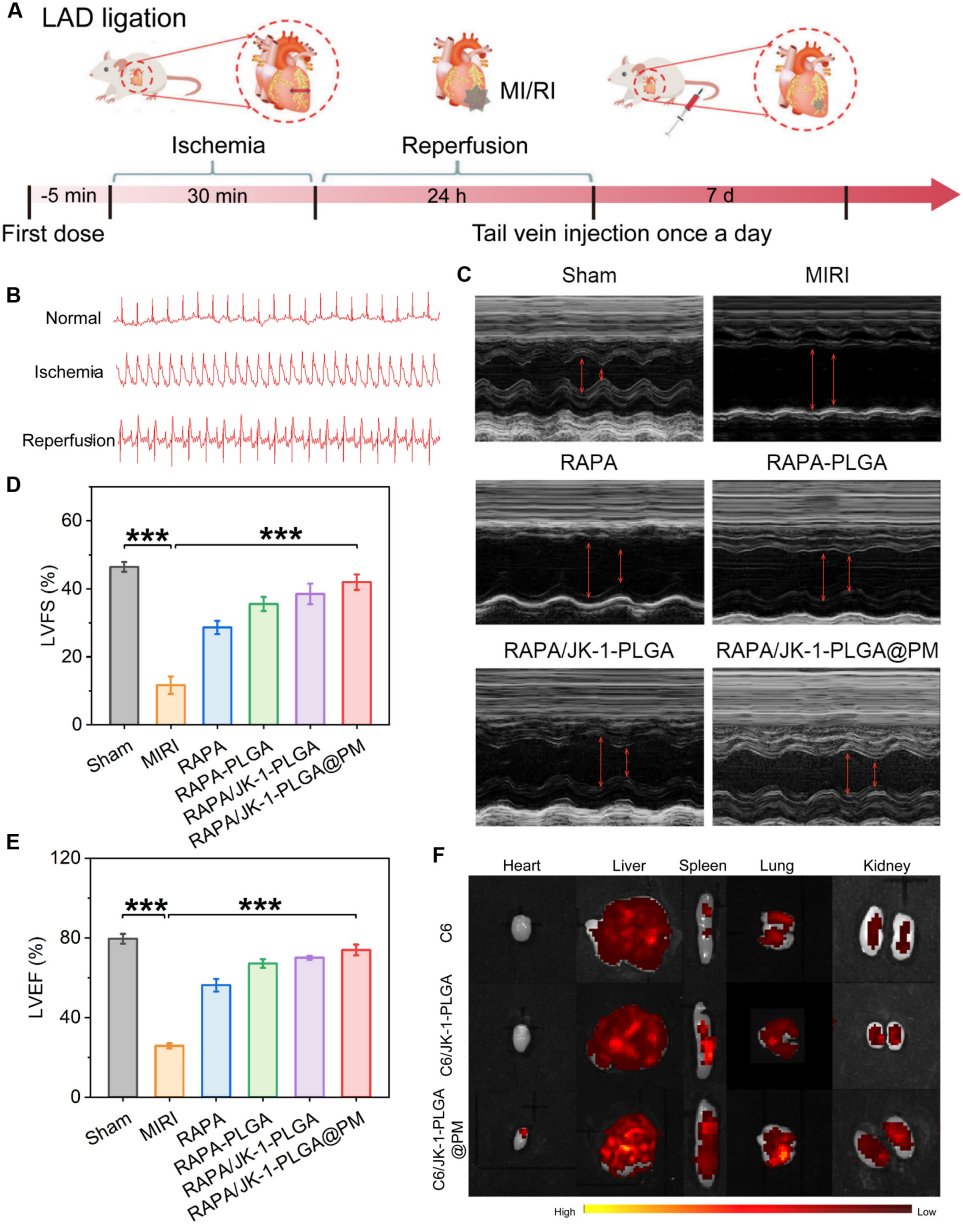
(A) Schematic diagram of in vivo experiments in the MIRI model. (B) Electrocardiogram of rats before and after modeling. (C) Echocardiographic imaging was performed on postoperative 7 days. Indexes of cardiac function of LVEF (D) and LVFS (E) were evaluated by echocardiography at different time points after the operation. (F) Fluorescence images of the heart and others major organs examined at 24 h after injection. Statistical analysis was performed using one-way ANOVA. When ***P < 0.001, **P < 0.01, and *P < 0.05, the difference was statistically significant. Data were shown as means ± SD (*n* = 3).

The mouse model of MIRI was constructed by ligating the left anterior descending coronary artery. After 7 days of administration, a series of experiments were conducted to investigate the in vivo therapeutic effects of RAPA/JK-1-PLGA@PM (Fig. 6A). The successful establishment of MIRI model validated by the real-time electrocardiogram was shown in Fig. 6B. The therapeutic efficacy of different preparations on cardiac function after I/R surgery was estimated by echocardiography. As displayed in Fig. 6C, the ventricular wall lost contractility, and the ventricular cavity was significantly enlarged in the MIRI group, compared with the control group. No significant abnormality in the left ventricle function was observed in mouse hearts treated with RAPA/JK-1-PLGA@PM, which manifested as a negligible change in the expansion of chamber dimensions and ventricular wall motion. Clinically, the cardiac function was often represented by the left ventricular (LV) systolic function, mainly evaluated in terms of the ejection fraction (LVEF) and fractional shortening (LVFS). As shown in Fig. 6D and E, MIRI was associated with a pronounced reduction in LV systolic function, expressed as the significant reduction of LVEF and LVFS compared with those in the Sham group. Compared to the MIRI group, treatment with RAPA/JK-1-PLGA@PM improved cardiac ejection function, observed by an increase in LVEF and LVFS, which almost returned to normal levels. These findings demonstrated that cardiac function was effectively improved in MIRI rats after treatment with RAPA/JK-1-PLGA@PM.

To investigate whether RAPA/JK-1-PLGA@PM could target the infarcted heart, the fluorescently labeled preparations were intravenously injected into mice before MI. The biological distribution of different preparations in the infarcted heart and major organs was determined using ex vivo fluorescence imaging (Fig. 6F). Compared to NPs without coated PM, the heart in the RAPA/JK-1-PLGA@PM group showed significant fluorescence intensity, suggesting that a large number of NPs accumulated in the apical area of MI. RAPA/JK-1-PLGA@PM was rarely accumulated in the liver, spleen, kidneys, and lungs after intravenous injection compared to other groups. The results indicated that RAPA/JK-1-PLGA@PM could specifically and effectively target the infarcted heart, with minimal off-target distribution in other organs. The designed nanoplatform relied on the natural infarct-homing ability of PM based on the binding of P-selectin on PM to PSGL-1 on monocytes to achieve active targeting for the injured myocardium [27]. Importantly, the ability of RAPA/JK-1-PLGA@PM to actively target the damaged myocardium supported the precise and sustained release of H2S to enable its physiological role.

**Fig. 7.**
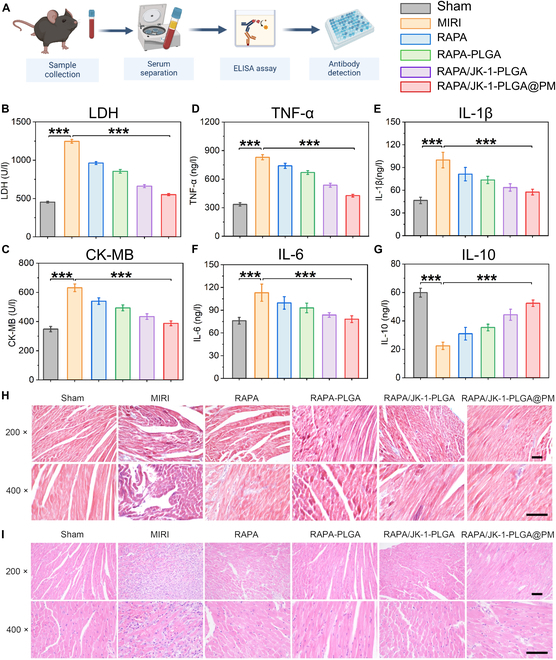
(A) ELISA experimental procedure flowchart. Cardiac levels of CK-MB (B) and LDH (C) in the serum of MIRI mice. Cytokine levels of TNF-α (D), IL-1β (E), IL-6 (F), and IL-10 (G) in the serum of MIRI mice. (H) H&E staining and (I) Masson staining of MIRI mice treated with different formulations. Scale bar, 200 nm. Statistical analysis was performed using one-way ANOVA. When ***P < 0.001, **P < 0.01, and *P < 0.05, the difference was statistically significant. Data were shown as means ± SD (*n* = 3).

Then, we investigated the regulatory effect of RAPA/JK-1-PLGA@PM on the myocardial injury in a mouse model of MIRI by ELISA (Fig. 7A). Compared with the Sham group, the marked increase of CK-MB and LDH concentrations in serum was observed to be induced by MIRI. The levels of CK-MB and LDH in serum reduced to varying degrees after treatment for all groups, compared to the MIRI group. The lowest concentrations of CK-MB and LDH were found in MIRI mice injected with RAPA/JK-1-PLGA@PM, further demonstrating the cardioprotective effect of RAPA/JK-1-PLGA@PM (Fig. 7B and C). After percutaneous coronary intervention in MIRI patients, ROS bursts activated caspase-1 and expression of potent pro-inflammatory cytokines, such as IL-1β and IL-6 [1]. RAPA/JK-1-PLGA@PM could also significantly inhibit the production of pro-inflammatory cytokines (TNF-α, IL-1β, and IL-6) and promote the excretion of anti-inflammatory cytokines (IL-10) compared with the Sham group (Fig. 7D to G). Taken together, consistent with the results of in vitro experiments in H/R cardiomyocytes, RAPA/JK-1-PLGA@PM could not only reduce myocardial injury but also reprogram the pro-inflammatory into anti-inflammatory state, thereby promoting the transfer into reparative immune microenvironment in vivo. In summary, these results demonstrated the advantages of using RAPA/JK-1-PLGA@PM for the treatment of MIRI. Gas therapy with exogenous delivery of H_2_S could limit cardiac damage and maintain cardiac function after I/R injury [42]. Continuous release of H_2_S and effective targeting of biomimetic nanoplatform could fundamentally improve the curative effect of the MIRI.

### Inhibition of the pathological remodeling

MIRI is accompanied with tissue damage, cardiac fibrosis, and cardiomyocyte apoptosis. The process of I/R injury inevitably led to a massive loss of cardiomyocytes, leaving the cells terminally differentiated and unable to regenerate. Consequently, cardiac dysfunction and the ensuing compensatory fibrosis culminated in pathological cardiac remodeling [43]. Histological assays were performed to investigate the effect of RAPA/JK-1-PLGA@PM on cardiac remodeling after MIRI. The Masson trichrome staining of myocardial tissue for all groups of MIRI mice is shown in Fig. 7H. For the Sham group, almost no collagen proliferation was found in the myocardial tissue, and the morphology of cardiomyocytes was normal and orderly. On the contrary, the MIRI group had severe fibrosis in the myocardial tissue, with obvious large-scale collagen deposition. Compared with the MIRI group, cardiomyocytes were relatively regularly arranged and myocardial fibrosis and collagen deposition were reduced in all groups after treatment. In particular, a significant reduction in collagen deposition and fibrosis of the myocardial tissue was observed in the RAPA/JK-1 PLGA@PM group. Furthermore, the H&E staining results of myocardial tissue in MIRI mice are shown in Fig. 7I. Myocardial tissue fibers were dense and clear, with normal cardiomyocyte structure, and almost no infiltration of inflammatory cells in the Sham group. However, the myocardial tissue fibers were broken, and the boundaries of cardiomyocytes were blurred, with obvious infiltration of a large number of inflammatory cells in the MIRI group. After the treatment of free RAPA, RAPA-PLGA, RAPA/JK-1-PLGA, RAPA/JK-1-PLGA@PM, the texture of myocardial fibers was relatively clear, arranged in a regular manner, and the degree of inflammatory infiltration was reduced. Compared with the RAPA group, the myocardial fiber rupture and inflammatory cell infiltration were significantly reduced in the RAPA/JK-1-PLGA@PM group. Consistent with the elevation in cardiac function, cardiac morphology was also improved by RAPA/JK-1-PLGA@PM. Hence, histological results demonstrated that RAPA/JK-1-PLGA@PM could inhibit myocardial fibrosis and improve myocardial remodeling by effectively releasing H_2_S.

## Conclusion

MIRI has an important negative impact on the therapeutic effects of revascularization therapy, thereby posing a challenge to the development of effective therapeutic drugs [1]. H_2_S, recognized as one of the gasotransmitters, has been shown to exert an infarct-limiting effect on MIRI. Elevated H_2_S levels protect against myocardial damage by promoting the transition to anti-inflammatory M2-type macrophages, relaxing blood vessels, stimulating angiogenesis, and limiting mitochondrial ROS generation [17]. However, off-target or even toxic effects are highly likely, with the H_2_S burst achieved due to the fact that H_2_S has a narrow therapeutic window. In order to gain access to controllable H_2_S release, JK-1 was used as an H_2_S donor, which produce H_2_S only at acidic pH [44]. Due to JK-1 being a small molecule that lacks targeting during intravenous injection, the development of the more advanced delivery system to prolong the H_2_S release and control its levels in vivo is required.

In this work, we created ischemic myocardium-targeting, H_2_S-controlled releasing NPs capable of being delivered by intravenous injection and specifically accumulating in the ischemic myocardium (Figs. 1 and 5F). NPs had RAPA/JK-1-PLGA complex as core and PM as shell. PLGA has a high molecular weight and can substantially decrease the diffusion rate of RAPA and JK-1 when loaded with them. JK-1 has the pH-sensitive property to respond to the release of H_2_S gas in the inflammatory environment of ischemic myocardium (Fig. 2F and G). This ensures that no H_2_S gas escapes to release into other organs or tissues. NPs injected into the bloodstream can be transported by monocytes to ischemic myocardium by mimicking the binding interaction of P-selectin on the PM with PSGL-1 on monocytes. Engineered biomimetic cell membranes have been widely used for precise targeted drug delivery [45,46]. In this study, PM-coated PLGA NPs were used for the delivery of JK-1 and RAPA. In addition, NPs showed good biocompatibility and hemocompatibility with minimal off-target accumulation in major organs.

We tested the therapeutic effect of RAPA/JK-1-PLGA@PM in a mouse model of MIRI. The study confirmed that MIRI occur with impaired cardiac function. After treatment, RAPA/JK-1-PLGA@PM significantly promoted the recovery of cardiac function and reduced the infarcted myocardial scar area, which in turn reduced myocardial injury and prevented the occurrence of myocardial fibrosis (Figs. 5 and 6). In addition, in vitro cellular experiments confirmed the effect of NPs on apoptosis and release of inflammatory factors in cardiomyocytes (Fig. 4). The controlled release of H_2_S could protect cardiomyocytes from oxidative damage and attenuate inflammation.

The H_2_S-releasing NPs developed in this work are better than those H_2_S-releasing systems based on inorganic donors and GYY4137. First, our NPs achieve intelligent pH-responsive release. Second, our engineered biomimetic NPs are safer and more biocompatible, and the targeting allows for a lower dosage of the drug to be administered. Third, our system does not suffer from a sudden release of H_2_S compared to those based on inorganic donors of Na_2_S and NaHS, which do not cause a sharp and short-lasting increase in H_2_S levels in vivo, producing toxic side effects [47].

We acknowledge that there were also some limitations in this study. First, this work was limited to murine studies, which may not be fully representative of human pathophysiology. Future studies using large animals will help to develop the translational potential of this technology. The NP concentration and injection volume need to be optimized for different animal models. Furthermore, although we have shown that RAPA and H_2_S synergistically significantly improve cardiac function and rescue damaged myocardium in the MIRI model, more detailed studies of the effects of H_2_S on injured myocardium are needed. In future studies, we plan to deliver H_2_S alone and investigate the effect for the treatment of MIRI.

In the present study, we successfully developed the engineered biomimetic NPs (RAPA/JK-1-PLGA@PM) for the treatment of MIRI based on gas therapy. RAPA/JK-1-PLGA@PM achieved active targeting of the heart after I/R injury by exploiting the natural infarct-homing ability of PM. The pH-sensitive property of JK-1 realized the precise release of H_2_S in the injured myocardial region, which played a critical role in MIRI treatment. After being released from RAPA/JK-1-PLGA@PM in damaged myocardium, H_2_S inhibited apoptosis, altered the inflammatory microenvironment, and attenuated myocardial injury. It subsequently synergized with the anti-inflammatory effects of RAPA to improve functional recovery of the infarcted heart and prevent pathological tissue remodeling. Accordingly, we believe that the designed nanoplatform offered a promising mentality for the clinical translation of gas therapy and a more effective therapeutic strategy for MIRI. Given the high outcome of H_2_S for cardiovascular and reperfusion injury aspects, we could envisage RAPA/JK-1-PLGA@PM for a wide range of applications in MIRI and other cardiovascular diseases.

## Data Availability

Data supporting the results of this study can be obtained from the corresponding author upon reasonable request.
